# On Cramp or Spasm

**Published:** 1853-07

**Authors:** P. J. Murphy

**Affiliations:** Stone-street


From the London Lancet.

*On Cramp or Spasm.* By P. J. MURPHY, M. D.

CRAMP is a sudden, involuntary, complete, and painful contraction of a muscle. All the voluntary and some of the involuntary muscles are liable to these contractions. Spasms is the term usually employed when this painful affection seizes on an involuntary muscle; of the latter class the most common sufferers are the heart and the uterus; the uterus can suffer spasms when enlarged only. It is questionable whether muscles of canals, or even those of the bladder, can suffer to such extent from spasmodic action as demands medical treatment.

The involuntary contractions of muscles may vary in degree, from the painless form of subtultus tendinum or chorea, to the excruciating tortures of tetanus or epidemic cholera. The remote causes are various and dissimilar—irritation of the spinal chord; of the medulla oblongata; of the peripheral extremity of a nerve, mechanical or pathological; disease within the cranium, or a poison, as ergot of rye, circulating through the vessels. Involuntary contractions, attended with insensibility, are termed convulsions. The observations here refer solely to what may be called local cramp or spasm, originating in, and restricted to one muscle, as observed in the gastrocnemius; in the uterus, as after-pains; and to that alarming spasm of the heart, angina pectoris.

*Cause.*—The immediate cause of local cramp would appear to be *a languid circulation in the veins which traverse the substance of a muscle.* This theory will explain why cramps attack most frequently the voluntary muscles furthest from the organs of circulation—the feet and legs; why they are more common in cold weather; why the principal sufferers are females, especially those with weak pulse, pale countenance, and chilly surface, and therefore why they are so seldom absent in chlorosis. It will also explain why the seizure is most usual in the horizontal position, when muscular action, so favorable to the circulation, has ceased. Nearly one half of the young, growing females, with a tendency to chlorosis, suffer from this annoying complaint; and it is remarkable, that although we have many cases where the cramps are limited to the muscles below the knee, we meet with none where the remark will apply to those above the knee. No inquiry being made about this symptom, it is seldom mentioned to the physician, and yet it is almost pathognomonic of an enfeebled action of the heart, from which the anemic headache springs. When this affection is complained of, whether in the aged or the young, no matter of what sex, the necessity for chalybeates is indicated. Dr. Brady makes one proper exception—in cases of pregnant females, but the cause of their languid circulation is mechanical, and therefore, not remediable by medicine. It may very naturally be asked, if a feeble and delicate constitution predisposes to cramp, why is it not an attendant on the last stages of diabetes, phthisis, and other exhausting diseases? Because those diseases produce hectic fever, and hectic is accompanied with a pulse so rapid as to be incompatible with a slow, venous circulation. Nor does it supervene in debility after profuse hæmorrhage; the rapid pulse of reaction and the half empty vessels affording a satisfactory explanation of its absence. The severest cramps we witness from arrest of circulation in the veins of muscles, are those which add so much to the horrors of epidemic cholera, for these cramps, although general, in an exact sense must be considered local, no muscle contracting until the blood in its own veins is arrested. It is an error to attribute the cramps of epidemic cholera to a poisonous quality of the disease, and the recollection that our own autumnal cholera is accompanied with similar cramps should disabuse us of this mistake.

In neither form of cholera do cramps supervene, until the venous circulation through the muscles is almost impeded from the density of the blood, a natural consequence of the exudation of its serous portion. No person can doubt that in epidemic cholera the circulation is from this cause almost arrested; for on puncturing a vein, the blood trickles down slowly, thick and dark as tar. Fatal cases of Asiatic cholera, it is true, are recorded, where few or no evacuations took place, yet cramps, although not violent, were noticed. Such cases might favor the opinion that the cramps depended on the inhalation of a specific poison, did not the post-mortem examination discover the exuded serum still in the intestinal tube, consequently the cramps were present, but less severe than where the exuded serum was too abundant to be retained in the intestines.

That there are poisons capable of coagulating the blood in its vessels, and that coagulation is attended with muscular contractions, cannot be denied. Dr. Pereira, in his last edition of "Elements of Materia Medica," quotes the *Journal de Chimie Medicale*, to show the physiological effects of bromide of potassium. Thirteen grains, dissolved in water, and injected into the jugular vein of a dog, coagulated the blood, and caused convulsions and death in a few minutes. But the same medicine taken into the stomach in larger doses produced vomiting only. As a clinical fact in English cholera, it is interesting to observe how much sooner the cramps cease when the stomach does not reject fluids; for the veins are soon refilled, and thus the proportion between the crassamentum and serum is quickly restored. The so-called secondary fever of Asiatic cholera arises solely from congestion of the venous system generally, and this congestion is peculiar, the fault existing in the blood itself being too thick, from having parted with its serum, to be influenced by the action of the heart. Rigors, or rapid involuntary contractions of muscles, are seen after exposure to cold, at the commencement of most fevers, and in the first stage of ague; for the first stage of these diseases is characterized by the desertion of blood from the superficial to the deep-seated veins, and these rigors or contractions are salutary; for by compressing the deep veins, the blood is restored to the superficial vessels, causing the phenomena of reaction. In fevers there can be nothing more alarming than the absence or prolonged delay of rigors. When scarlatina threatens to be unfavourable within twenty-four or thirty-six hours from its invasion, there are no rigors, for the muscles seem to have lost this salutary contractile power. A cramp may therefore be regarded as a painful but useful action of a muscle to assist in the circulation of venous blood.

*Angina Pectoris.*—No person can witness a paroxysm of this disease in a fellow-creature without a feeling of sympathy. The pain is most acute, and the alarm and distress pitiable. It is admitted that angina is a spasm of the heart, but the cause of the spasm has been attributed to almost every disease to which the heart is liable—ossification of the coronary arteries, disease of the valves, hpertrophy of the vetricles, and fatty degeneration of its substance, and certainly it has co-existed with most of those diseases; but yet, on the other hand, almost every form of heart-disease is daily witnessed, yet happily unaccompanied with angina. There is, however, no well authenticated report of angina in an uncomplicated case of adhesion of the pericardium, and it seems anatomically impossible. Simple ossificaton of an artery is not a cause of cramp; there is no cramp attends Pott's mortification, for ossification rather cuts off the supply of blood, and therefore the veins must be more or less empty.

If we admit, and it can scarcely be denied, that the arrest of venous blood in a muscle is a cause of cramp, we have a very plausible explanation of the proximate cause of angina, and why it may arise from almost every disease of the heart, and yet be absent in diseases apparently similar, for it is only when there is an impediment in the circulation of the coronary veins that the phenomena of angina is produced. Practitioners must have met a modified form of angina which occasionally attends severe cases of chlorosis, or when the circulation is languid from "relachement du cœur." Sudden pain is felt after exertion in the cardinac region, and now and then attended with pain on the inside of the left arm. This pain is quite distinct from the neuralgia of the eighth or ninth intercostal nerve, so common in the hysterical female, and from which the male sex never suffers, while it is not exempt from this angina, especially if the action of the heart be depressed by excess in smoking tobacco.

*Ergot of Rye* coagulates the blood. After parturition, the veins and sinuses of the uterus being full of blood, while the circulation is more languid, coagulation easily takes place; hence the muscle contracts to expel the blood from its substance. It is an erroneous idea to imagine that the ergot has a specific action on the uterus. On the unimpregnated uterus it has no action. It has utterly failed me as an emmenagogue. Even if pregnancy be of four months' duration no perceptible action is manifested, for in a few such cases I have inadvertently prescribed it, deceived by the character and representations of the patient. That it has the power of coagulating the blood is shown by mortification of the parts most distant from the heart, being the effect when it has been taken for any length of time. It is surprising how strongly impressed medical men are with the idea that after-pains are occasioned solely by clots in the womb. Before the adoption of this opinion it should be asked how it is that the uterus, which tolerated for nine months the gradually increasing bulk of the fœtus, could be thrown into spasms from the presence of a small quantity of blood. Hydatids and moles will form, and distend it to an immense size, and yet it will be passive under their irritation.

Many facts countenance the idea that the primary cause of parturition is the coagulation of the blood in the sinuses, which is necessarily followed by muscular contraction. Ergot of rye coagulates the blood, contraction follows; small-pox kills the fœtus, the circulation ceases, contraction follows, in epidemic cholera the liquor amnios is removed, death then follows, and contractions supervene. The unimpregnated uterus is not liable to spasm.

*Dysmenorrhœa.*—The anatomy of the unimpregnated uterus displays its muscular fibres in a complete state of contraction; the cavity is almost nominal, and therefore a spasm or cramp of this organ is impossible. Dysmenorrhœa is not spasm, the pain is continuous, and women describe the pain as quite dissimilar from that of labour. As in such cases the menstrual fluid escapes with difficulty, the pain is probably that of distention, owing to the os tincæ being narrowed. Dr. M'Intosh, of Edinburgh, relieved his patients by introducing a metallic sound, proving the correctness of his theory; and another proof is, that mothers are seldom if ever annoyed with this complaint, although previously to parturition they were monthly martyrs.

We hear frequent mention of spasms of the sphincters of the rectum and bladder, and as their veins are very small the retardation of blood could not be the cause of these local cramps. But contraction is the normal state of a sphincter, and the contraction is so complete as to resist the escape either of urine or gas; closer contraction seems impossible, rendering the idea of spasms improbable. Since the discovery that the severe pain at the anal orifice when emptying the rectum depends on ulcers and fissures of that part, spasmodic stricture of the rectum is never heard of. Sphincters have no self-dilating power; they would therefore remain permanently contracted were it not for the mechanical action of the urine and fæces escaping.

The pain of cramp arises not from muscular contraction, but from the severe pressure on the nerves of sensation which traverse a muscle. Voluntary contraction is incapable of producing pain. Paraplegia and other diseases would furnish me with more abundant proofs, but enough has been said to let the correctness of these remarks be tested.

Stone-street, May, 1853.

